# Accurate simulations of magnetic excitations in the neutron simulation package *McStas*

**DOI:** 10.1107/S1600576726002414

**Published:** 2026-04-30

**Authors:** Silas B. Schack, Kristine M. L. Krighaar, Emma Y. Lenander, Kim Lefmann

**Affiliations:** a Nanoscience Center, Niels Bohr Institute, University of Copenhagen, Denmark; b Institute of Physics, École Polytechnique Fédérale de Lausanne (EPFL), CH-1015 Lausanne, Switzerland; Technical University of Denmark, Denmark

**Keywords:** spin waves, neutron scattering, ray-tracing simulations, *McStas*, altermagnetism

## Abstract

An algorithm for the accurate simulation of neutron scattering from magnetic excitations has been developed and implemented as new component for the neutron ray-tracing software *McStas*. The component SpinWave_BCO simulates inelastic neutron scattering from ferro-, antiferro- and altermagnetic excitations in a body-centred orthorhombic crystal structure, where the dispersion relation and scattered neutron intensities are derived using linear spin wave theory. Data from a simulated triple-axis spectrometer with an extremely high resolution have been verified by direct comparison with theory and by comparison with data simulated using the package *SpinW*. The component serves as a proof of concept for the implementation of a more general linear spin wave component in *McStas*.

## Abstract 2

We present an accurate algorithm for simulating neutron scattering from spin waves in the ray-tracing package *McStas*.

## 1. Introduction

Neutron scattering is an essential tool for investigating the structure and dynamics of condensed matter, being sensitive to both the nuclei and the magnetic moments in a sample (Boothroyd, 2020 ▸). In a neutron scattering experiment, one generally measures both the neutron energy and momentum transfers: 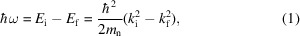

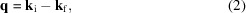
where 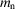 is the neutron mass, ℏ is Planck’s constant, *E* is the neutron energy, **k** is the neutron wavevector, and the indices ‘i’ and ‘f’ denote the initial and final neutron states, respectively.

Since access to neutron scattering beam time is limited, much instrumental design and preparation is carried out by neutron ray-tracing simulations. Here, virtual experiments (Lefmann *et al.*, 2008 ▸) on digital twins of neutron instruments can provide valuable insight into instrument performance, experiment feasibility, experiment resolution, multiple scattering effects and signal-to-background relations. In addition, much simulation work is performed for the analysis and design of novel neutron instrumentation, *e.g.* at the upcoming European Spallation Source, ESS (Andersen *et al.*, 2020 ▸).

One of the most used ray-tracing tools is *McStas*, a package originating from the 1990s (Lefmann & Nielsen, 1999 ▸) and continuously upgraded since. In the package, neutron instruments are assembled by positioning tailor-made or system-provided components (Willendrup & Lefmann, 2020 ▸). Among many existing system components, *McStas* contains a number of model neutron scattering samples, providing incoherent scattering, small-angle scattering, reflectivity and crystal diffraction (Willendrup *et al.*, 2006 ▸; Willendrup & Lefmann, 2021 ▸; Christensen *et al.*, 2026 ▸). However, the number of inelastic scattering samples in *McStas* is at present very limited, the only examples being a flat inelastic mode (akin to a crystal electric field level), a single quasielastic Lorentzian line and a one-branch phonon sample. This seriously limits the breadth of inelastic virtual experiments that can be performed in *McStas*.

A component for magnon scattering, called Magnon_bcc, currently exists in the *McStas* component library and was written by some of us. However, this component is limited in scope and has never been fully developed or validated. In addition, we note that the simulation package *McVine* contains simulations of general dispersion relations, although the full algorithm is to our knowledge not published (Lin *et al.*, 2016 ▸; Lin *et al.*, 2019 ▸).

In this work, we remedy this situation by presenting the new *McStas* component SpinWave_BCO, which calculates and simulates the spin waves of a ferromagnet or a two-sublattice antiferromagnet in the body-centred orthorhombic structure. The component has been tested in a simple virtual experiment using a thermal triple-axis spectrometer with an unrealistically good resolution in both 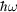 and **q**. The simulated dispersion and intensity match convincingly both with analytical calculations and with the output of the package *SpinW* (Toth & Lake, 2015 ▸). We expect this component to be useful as a test of instrument design as well as for detailed analysis of spin wave data. The component can be readily upgraded to accept a more general lattice geometry and interaction pattern.

## 2. Spin wave theory

We here describe the theory of quantized spin waves (magnons), also known as linear spin wave theory, that we have used as a basis for our simulations. Although this topic has been known for more than half a century, spin wave theory comes in different variations, *e.g.* how to specify anisotropy constants and infamous factor 2 variations in definitions of the interaction constants. For these reasons, we deem it necessary to document exactly which equations lie behind our simulations. The main focus will be on the antiferromagnetic spin waves in the body-centred orthorhombic structure. However, the component will in addition simulate ferromagnetic spin waves in the same crystal structure, so a short introduction to the ferromagnetic case is also given.

The magnetism in the system of interest is governed by Heisenberg interactions with coupling strength 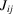, uniaxial anisotropy parametrized by the constant *D* and an applied magnetic field with field strength *B*. For simplicity, we here align both the easy axis and the field along the crystallographic *c* axis (the **z** direction). The field is applied to stabilize the spins (**s**_*i*_) in the positive **z** direction. The system is thus described by the Hamiltonian 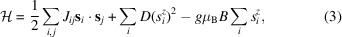
where 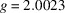 is the electron gyromagnetic ratio and 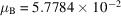
 meV T^−1^ is the Bohr magneton. When 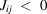 dominates, the system is said to be ferromagnetic, while 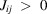 dominating leads to an antiferromagnetic system.

### 2.1. Ferromagnetic spin waves

The ferromagnetic (FM) case for 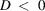 is relatively simple, as the eigenstate of the Hamiltonian equals the classical ground state, where all spins are oriented along the +**z** direction.

The magnetic dynamics of the system are found using linear spin wave theory, where the deviations from the ground state are assumed to be small. The Hamiltonian is written using the spin raising and lowering operators for spins at position 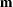: 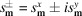. This is then reduced to second order by the Holstein–Primakoff transformation (Boothroyd, 2020 ▸): 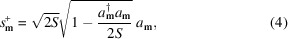

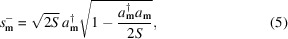

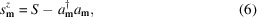
where *S* (unitless) is the spin value, and 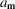 and its Hermitian conjugate are bosonic operators satisfying the commutation relation 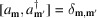.

**Figure 1:**
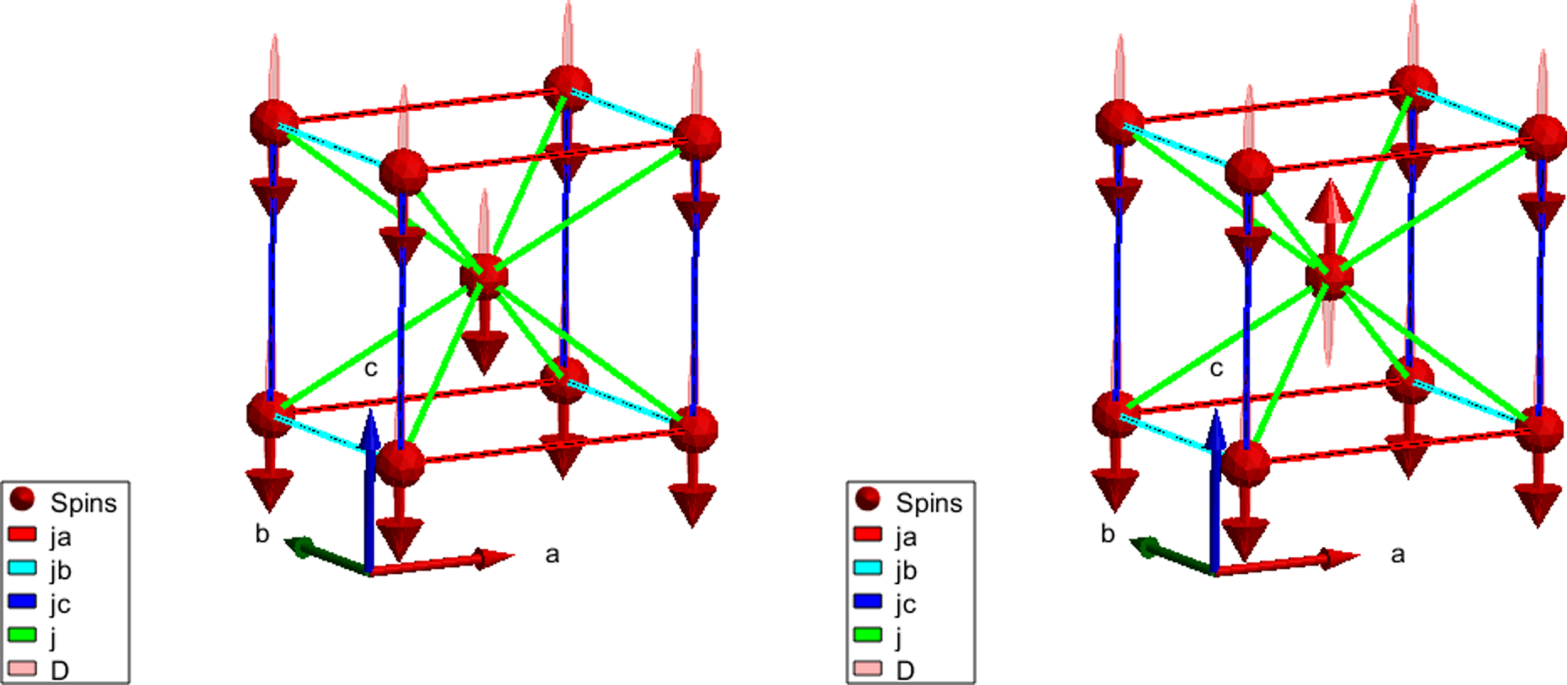
Magnetic structure and exchange parameters for the ferromagnetic (left) and antiferromagnetic (right) system. The plots are generated using *SpinW* (Toth & Lake, 2015 ▸).

Using a first-order expansion of the Holstein–Primakoff transformations in the Hamiltonian, and further Fourier transforming the bosonic operators, leads directly to the diagonal Hamiltonian 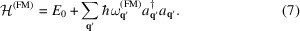
Here 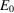 is the energy of the ground state and 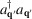 counts the number of magnons of wavevector 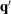 and energy 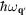, with 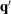 defined within the first Brillouin zone. The spin wave dispersion relation is given by 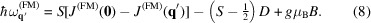
Here we have defined the Fourier transform of the coupling constants as 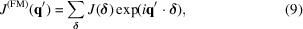
where 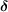 are vectors connecting one particular spin to its neighbours. We assume couplings 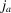, 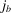 and 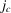 to the nearest neighbours along each crystallographic axis in our body-centred orthorhombic structure. In addition, we add couplings *j* to the spins in all body-centred positions. The system is shown in Fig. 1 ▸ (left). It is convenient to separate 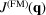 into two parts accounting for the interactions with the body-centred spins and spins along the crystallographic axes, respectively. We then obtain 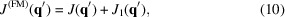
with 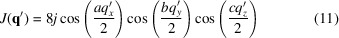
and 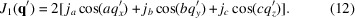
Neutron scattering from magnons happens at low temperatures primarily via the creation or annihilation of a single magnon. The partial differential cross section for unpolarized neutron scattering from single magnons in the ferromagnet is (Marshall & Lovesey, 1971 ▸)
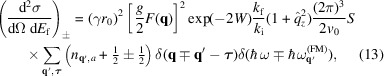
where 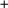 (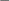) refers to the creation (annihilation) of a magnon with energy 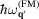. In the expression, 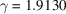 is the neutron gyromagnetic ratio, 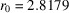
 fm is the classical electron radius, 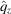 is the *z* component of a unit vector along the neutron scattering vector **q**, 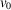 is the volume of the magnetic unit cell and 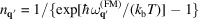 is the thermal occupation number of (bosonic) magnons at temperature *T*, with *k*_b_ the Boltzmann constant. The vectors **τ** are the reciprocal lattice vectors of a sublattice. Both the magnetic form factor, 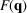, and the Debye–Waller factor, 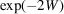, will be approximated as constants equal to 1.

### 2.2. Antiferromagnetic spin waves

In this section we describe the dynamics of spin waves in a classical two-sublattice antiferromagnet (the Néel state). The magnetic unit cell of such an antiferromagnetic (AFM) system is shown in Fig. 1 ▸ (right).

As with the ferromagnet, the magnetic dynamics are found using linear spin wave theory. In this case we perform the Holstein–Primakoff transformations for each sublattice, where the 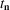 operators represent the spin operators of the ‘down’ sublattice rotated by 180° about the *x* axis: 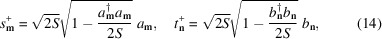

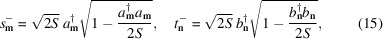

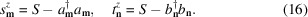
After expanding to first order and Fourier transforming to the operators 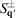 and 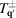, the resulting Hamiltonian can be diagonalized by a Bogoliubov transformation to bosonic operators 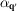 and 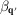 via
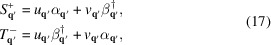
where 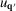 and 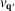 are real coefficients fixed by requiring that all operators satisfy the relevant commutation relations, *e.g.*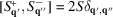 and 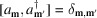. This leads to the final Hamiltonian 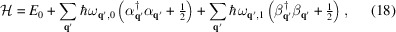
where 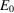 is the energy of the approximate ground state. 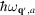 denote the two spin wave dispersion relations, which read (Marshall & Lovesey, 1971 ▸)
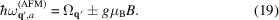

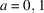
 is the mode index, which refers to the positive and negative signs of the Zeeman term, respectively, and 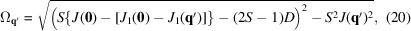
where the Fourier-transformed coupling constants are again given by equations (11) and (12).

The partial differential cross section for unpolarized neutron scattering from single magnons in an antiferromagnet is (Marshall & Lovesey, 1971 ▸)
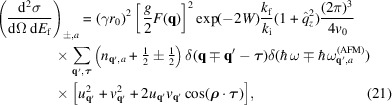
The factor in the last line of the equation is denoted the *coherence factor* and consists of the coefficients of the Bogoliubov transformation. The vector 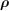 connects nearest-neighbour spins on different sublattices and is given by 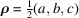 for the body-centred structure. One can show that given the condition for conservation of crystal momentum 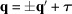 the following identity holds:
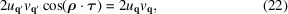
meaning that the coherence factor can be calculated directly from the neutron scattering vector 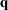. Using this, the coherence factor is given by
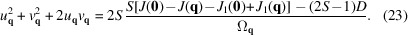
Since 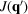 has a period of two Brillouin zones, as can be seen from equation (8), the coherence factor causes the scattered intensity to vary between different Brillouin zones, having the highest value when **q**′ is close to an antiferromagnetic ordering vector.

## 3. Implementation

We now describe the calculations implemented in the new component SpinWave_BCO in *McStas*. This component simulates the spin wave spectrum of the magnetic systems described above, incorporating both the dispersion relations (8) and (19) and the scattered intensity, which will be derived from equations (13) and (21).

*McStas* simulates neutron scattering experiments using Monte Carlo ray-tracing techniques. Neutrons are treated as classical rays defined by the components of their position, velocity and spin. To economize computing power, each ray is treated as a number of neutrons, with the equivalent neutron number given by a (non-integer) weight factor, *p*, assigned to each ray (Willendrup & Lefmann, 2020 ▸). When a ray interacts with a component, the ray parameters, including its weight, are subject to change. The weight factor is modified by the weight multiplier *w*, such that the weight factor after the interaction with component *j* is given by 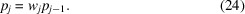
Scattering events are simulated using Monte Carlo sampling, where we denote the sampling probability of a given event *a* by 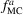. The weight multiplier 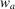 is then the ratio between the physical probability of that event 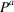, which is found from the cross section, and the sampling probability 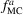 (Willendrup & Lefmann, 2021 ▸): 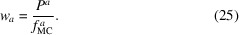
As an example, to simulate reflection in a neutron guide, one would only simulate reflected neutrons, meaning 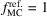. If *e.g.* the probability of reflection is 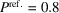, we would obtain a weight multiplier of 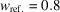.

For the SpinWave_BCO component, the Monte Carlo choices consist of (*a*) selecting a scattering direction, 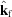, and (for the antiferromagnetic system) (*b*) selecting one spin wave mode out of 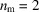 possibilities. The former is determined with a uniform distribution within a predefined solid-angle interval, 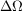. When a neutron scatters from an excitation following a dispersion relation 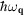, in this case a magnon in the chosen magnetic system, it must obey the kinematic constraint 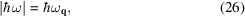
where the spin wave dispersion 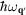 is now generalized to the neutron wavevector 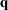 by periodicity. Via the kinematic constraint, the neutron fulfils one of the delta functions in equation (13) or (21). Given that the direction of 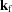 is already chosen, the kinematic constraint can be solved, giving 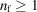 possible values of the final neutron speed 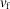 (Squires, 1977 ▸). These values of 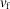 are determined, and a third MC choice (*c*) selects one of these values. This algorithm is essentially equivalent to MC simulation of phonon scattering, implemented in *McStas* through the components Phonon_simple and Phonon_PG and described by Davidsen *et al.* (2026 ▸). It is here derived that the weight multiplier of the component is given by 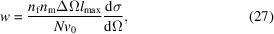
where 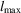 is the path length of that particular neutron ray inside the sample if it does not scatter, 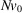 is the total volume of the sample and 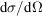 is the differential cross section. The differential cross section is defined as 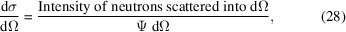
where Ψ is the flux of the incoming neutron beam. The cross section thus describes the total number of neutrons scattered into a particular direction, regardless of their energy (Booth­royd, 2020 ▸).

When searching for solutions to the kinematic constraint, the initial neutron velocity and the direction of the neutrons’ final velocity, 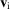 and 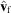, respectively, are kept fixed, while the neutron scattering vector and energy transfer are defined by the final neutron speed according to 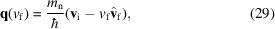

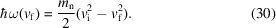
This transforms the kinematic constraint into a one-dimensional problem, easily solved by standard numerical techniques; for details of this procedure, see Davidsen *et al.* (2026 ▸). The differential cross section 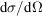 is calculated from 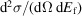 [equations (13) and (21) for the ferromagnet and antiferromagnet, respectively] by integrating over 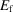. To do this, we utilize the relation 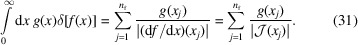
Here, 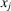 is the *j*th (out of 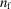) positive zero of 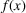, and we have defined 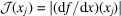. We can now perform the integration of the inelastic cross section, leading to the expression for the differential cross section for the creation (annihilation) of a single magnon with final speed 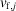 out of the 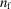 possible values. For the ferromagnetic case, this leads to 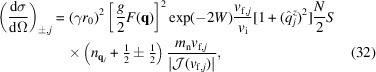
while for the antiferromagnetic case, the expression for mode *a* is 
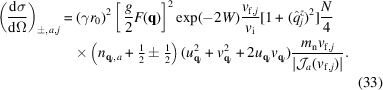
In the simulation, 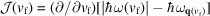 is found numerically for the selected value of 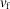.

The input parameters for the component are listed in Table 1 ▸.

The component takes the form of a cylindrical sample. When it is placed without rotation, the *a*, *b* and *c* axes lie along the *x*, *y* and *z* axes of the *McStas* coordinate system, respectively.

The main functionality of the component is listed in Algorithm 1:
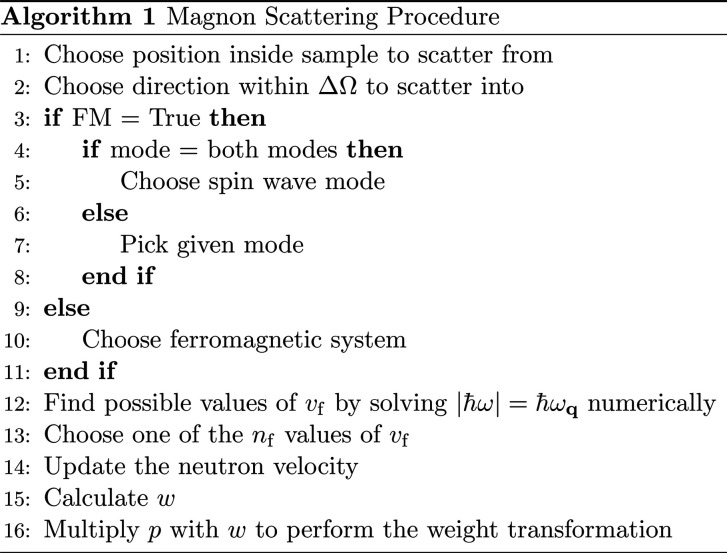
In these calculations, by far the largest computational power is expended in finding the relevant values of 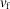 that simultaneously fulfil the dispersion relation and the kinematic constraint.

The component also conducts a number of checks of the calculated expressions to ensure that the input parameters lead to physically meaningful results. An error message is produced if the dispersion becomes negative or imaginary and if the coherence factor becomes negative when simulating an antiferromagnetic system.

These would probably be caused by the input parameters that do not correspond to the chosen ground state (FM or AFM). When simulating a ferromagnetic system, the value of *B* is forced to be positive (or zero), such that the applied field always stabilizes the ground state. For the antiferromagnetic system, the magnetic field strength is set to zero if it induces a spin-flop transition, *i.e.* if the dispersion for the low-energy mode becomes negative.

## 4. Validation

The component was validated by comparing theoretical calculations with simulated data. This includes a validation of both the simulated dispersion relation, by extracting the Hamiltonian parameters from simulated data, and the simulated absolute intensities.

The main focus was on the validation of the antiferromagnet. This was done by modelling the spin wave spectrum of MnF_2_. This material has previously been understood as a classical antiferromagnet, with the magnetic Mn^2+^ ion situated in a body-centred tetragonal lattice. The crystal and Hamiltonian parameters for MnF_2_ used in the simulations are listed in Table 2(*b*) ▸.

We employ a virtual thermal triple-axis spectrometer (TAS), operating with a fixed final energy of 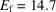
 meV. The instrument was previously used to test the Phonon_PG component and is described by Davidsen *et al.* (2026 ▸). The simulated instrument is tuned to have an unrealistically high resolution by reducing the sizes of the instrument components, such as the source, monochromator, sample and analyser. The energy FWHM of the instrument at the elastic line was found to be 0.08 meV using a simulation with the Incoherent sample component. Furthermore, the simulations do not include any sources of background or elastic scattering from the crystal. All of this aids in the comparison between the simulated datasets and the linear spin wave theory results. For the simulations, an initial number of 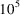 rays were used for each collected data point.

The data for the simulated dispersion were obtained as constant-energy scans along four paths in **q** space, each of which was confined to the (*h*0*l*) plane. Each scan was performed with a step size of 0.01 meV from 0 to 8 meV for the antiferromagnet and 0 to 13.2 meV for the ferromagnet. The sample temperature was set to 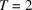
 K for all simulations, much lower than the ordering temperature of MnF_2_, 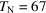
 K (Okazaki *et al.*, 1964 ▸).

We will start by showing that the ferromagnet produces the result expected from theory. To do this, we artificially set all exchange interactions to be FM (negative), keeping their magnitude the same as those for AFM MnF_2_. The exchange interactions are displayed in Table 2 ▸.

### 4.1. The ferromagnetic case

**Figure 2:**
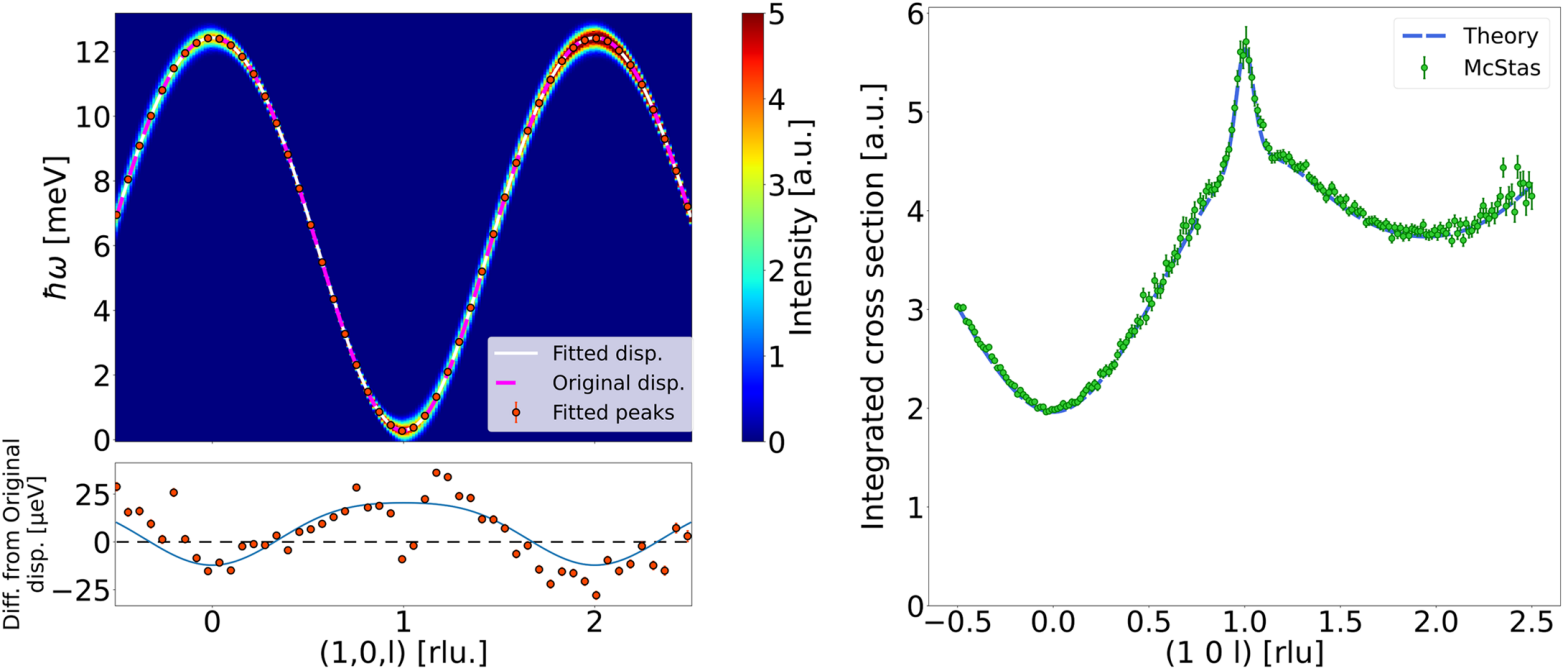
Simulated data for the ferromagnetic systems. (Top left) Simulated dispersion compared with theory. Red points show the fitted peak positions. The solid white line shows the dispersion calculated from the fitted parameters and the dashed magenta line shows the dispersion calculated from the original model parameters. (Bottom left) The difference between the fitted peak positions and the original dispersion (red points), and the difference between the fitted dispersion and the original dispersion (solid line). Ideally, they should be as close to zero as possible (dashed line). (Right) Integrated differential cross section compared with theory.

The ferromagnetic dispersion has been simulated along (10*l*). A magnetic field of 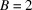
 T is applied to increase the size of the gap at the Brillouin zone centre. This is done to separate the spin wave creation and annihilation signals. A very small overlap between these two signals is still present, but the effect is relatively small. Gaussian line shapes were fitted to the constant-*q* scans to give a rough estimate of the intensity peak centres. The analytic expression for the dispersion given by equation (8) was fitted to the 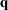 dependence of the fitted peak positions, and the resulting parameters were compared with the original model parameters to demonstrate the consistency of our method. Since the scan is along *l*, only 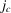, *j* and *D* were kept as free parameters in the fit (Table 3 ▸). The intensity data, fitted peak positions, and dispersions calculated from the fitted parameters and the original model parameters, respectively, can all be seen in Fig. 2 ▸ (top left). In Fig. 2 ▸ (bottom left) we show how the fitted peak positions and fitted dispersion (the dispersion calculated from the parameters extracted from the fit) deviate from the model dispersion. The deviations for both the points and the fitted dispersion are small. They generally follow each other as expected, except at **q** = (1, 0, 1), where mixing with the annihilation signals leads to lower energy values for the fitted peak positions.

Although the shape of the fitted dispersion is seen to match theory, the extracted parameters do not exactly match those used in the simulation; the value of the anisotropy constant *D* deviates by around 50%. However, given that *D* is very small and mainly determined by the data near the gap, where the creation and annihilation signals overlap, it is not surprising that this analysis exhibits minor deviations. For the antiferromagnetic case we show how a more in-depth analysis is able to reproduce the simulation parameters with a much higher accuracy.

In addition to fitting the dispersion, we compare the simulated relative intensities with those expected from theory. The intensities are first normalized to the incoming flux and then integrated along the energy transfer, which also removes the instrument-dependent energy broadening. The values from theory are normalized to the 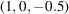 point of the data, and the comparison is shown in Fig. 2 ▸ (right). The simulated data are seen to reproduce the theoretical values with excellent agreement.

### 4.2. The antiferromagnetic case

For the antiferromagnetic system, we simulate constant-*q* scans from the classical antiferromagnet MnF_2_. The Hamiltonian input parameters were taken from Okazaki *et al.* (1964 ▸) and Nikotin *et al.* (1969 ▸) and are listed in Table 2(*b*) ▸.

**Figure 3:**
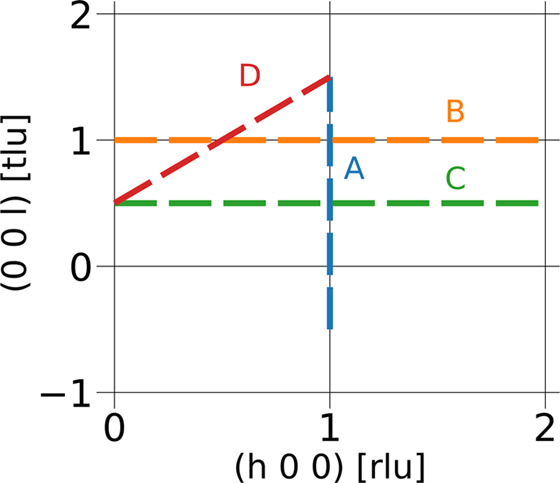
Map over scanned directions in reciprocal space for the antiferromagnetic case. Labels correspond to those in Fig. 5.

The dispersion was simulated along four directions in reciprocal space, as illustrated in Fig. 3 ▸.

**Figure 4:**
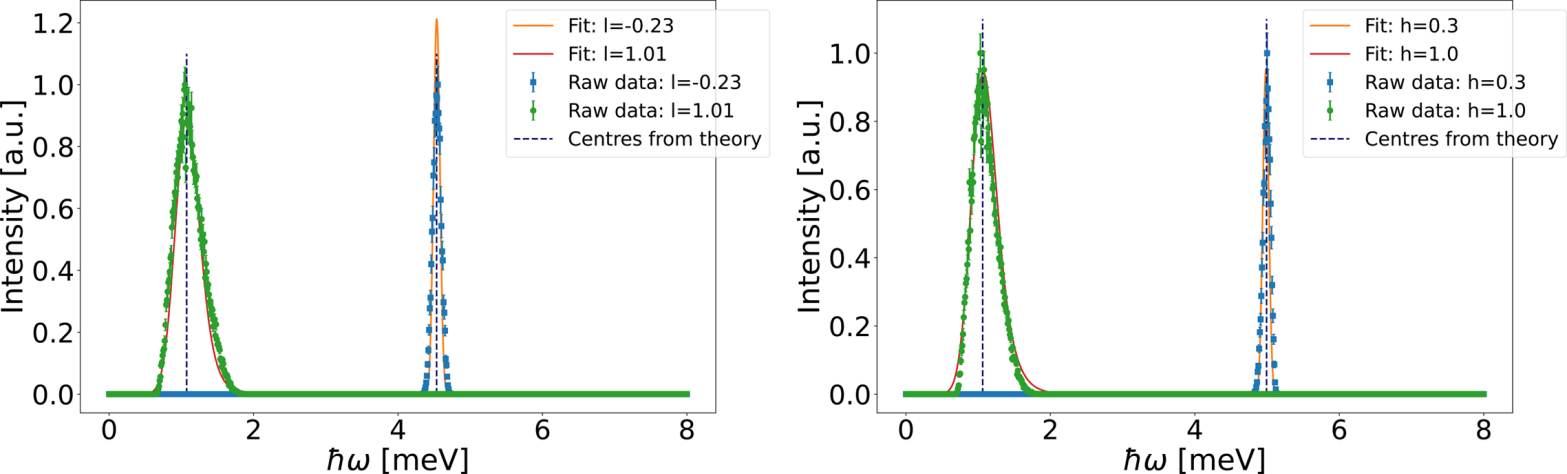
Examples of constant-*q* scans for the antiferromagnetic case (left) along (10*l*) and (right) along (*h*01). The solid lines show the fitted functions used to find the intensity peak centres and the dashed lines show the peak centres from theory.

As in the ferromagnetic case, the centre of each constant-*q* scan intensity peak was found by fitting Gaussian line shapes to the data, as illustrated in Fig. 4 ▸. Due to the instrumental resolution function, the shape of the intensity peaks varies between different values of **q**. A symmetric Gaussian function is used to fit most peaks. However, near the Brillouin zone centres, at the bottom of the dispersion, the resolution function makes the peaks highly asymmetric. For these scans, an approximate resolution convoluted gap function for TAS instruments [described by Lenander *et al.* (2026 ▸)] is used to fit the mode positions. In this function, it is assumed that the **q** resolution is coarse in two out of three directions, and at the gap, the dispersion follows a parabola, which are both valid in this setup. The function describes the asymmetric shape well; examples of the fits can be observed in Fig. 4 ▸ at low energies. Due to the extremely high energy resolution of the instrument and the assumption that the energy resolution is not correlated to the **q** resolution, the fitted mode positions are slightly underestimated (deviations less than 0.1 meV).

The analytical expression for the dispersion given by equation (19) is fitted to the **q** dependence of the fitted peak positions. We fitted all four datasets at once, where only the interaction parameters *j*, 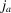, 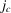 and *D* are allowed to vary. This gives us a direct comparison with the theoretical dispersion relation.

**Figure 5:**
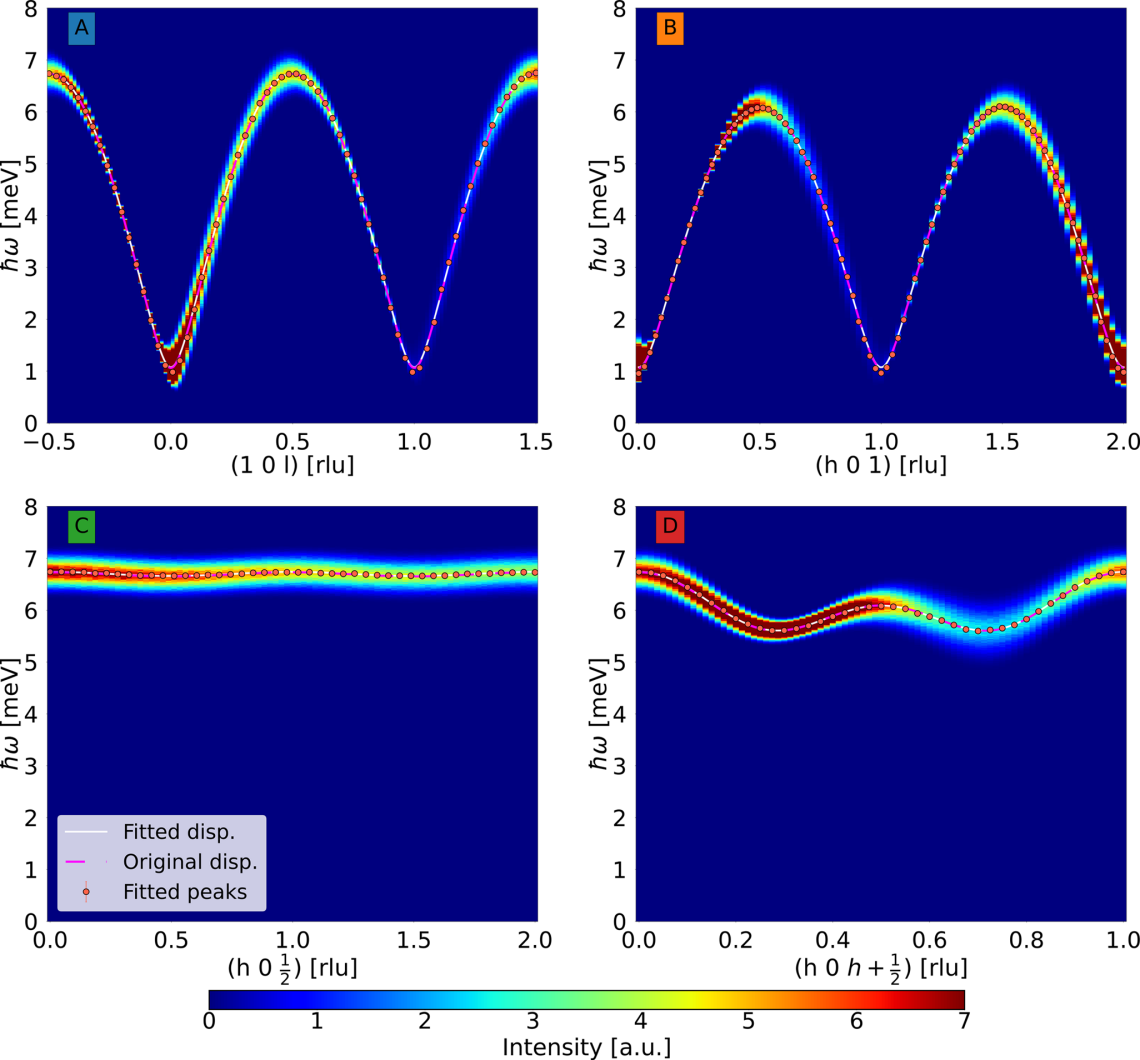
Simulated intensity data measured along four directions in **q** space. Points show fits to intensity peaks. The solid white line is the dispersion calculated from the fitted parameters and the dashed magenta line shows the dispersion calculated from the original model parameters.

In Fig. 5 ▸ we perform this comparison by displaying the raw intensity data, the peak positions obtained from the constant-*q* fits, and the dispersion relations calculated from the fitted parameters and the original model parameters, respectively.

The values of the fitted parameters are reported in Table 4 ▸, with the statistical uncertainties from the fits. The general agreement with the actual interaction values used as input to the component is excellent, with the largest deviation being only around 2.6% (for the small *D* parameter). This shows that our simulation method produces the correct dispersion and is self-consistent.

We will further validate the intensities simulated by the component used for the case of MnF_2_. This is done by recording the simulated differential cross section, obtained through equation (28), at several points on the dispersion curve. In the simulations, we directly recorded the incoming flux and the outgoing intensity by placing energy-sensitive monitors just before the sample and analyser in the TAS setup. The solid angle was calculated from known geometry parameters.

We compare these values with the results of spin wave theory through equation (33), as shown in Table 5 ▸. The agreement is generally excellent. The largest relative deviation is around 12% for the very weak annihilation signal: 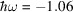
 meV. The deviation is around 6% near the low-intensity ferromagnetic point (1, 0, 1). All other deviations are on the order of 2% or lower.

The simulated intensities are also compared with a simulation of the dynamical correlation function performed using the MATLAB library *SpinW* (Toth & Lake, 2015 ▸). The datasets simulated along the (10*l*) direction were used for this. To compare the two simulated datasets, each was converted to quantities proportional to the inelastic cross section. The *McStas* intensities were normalized and integrated along the energy transfer, as was done for the ferromagnetic data.

**Figure 6:**
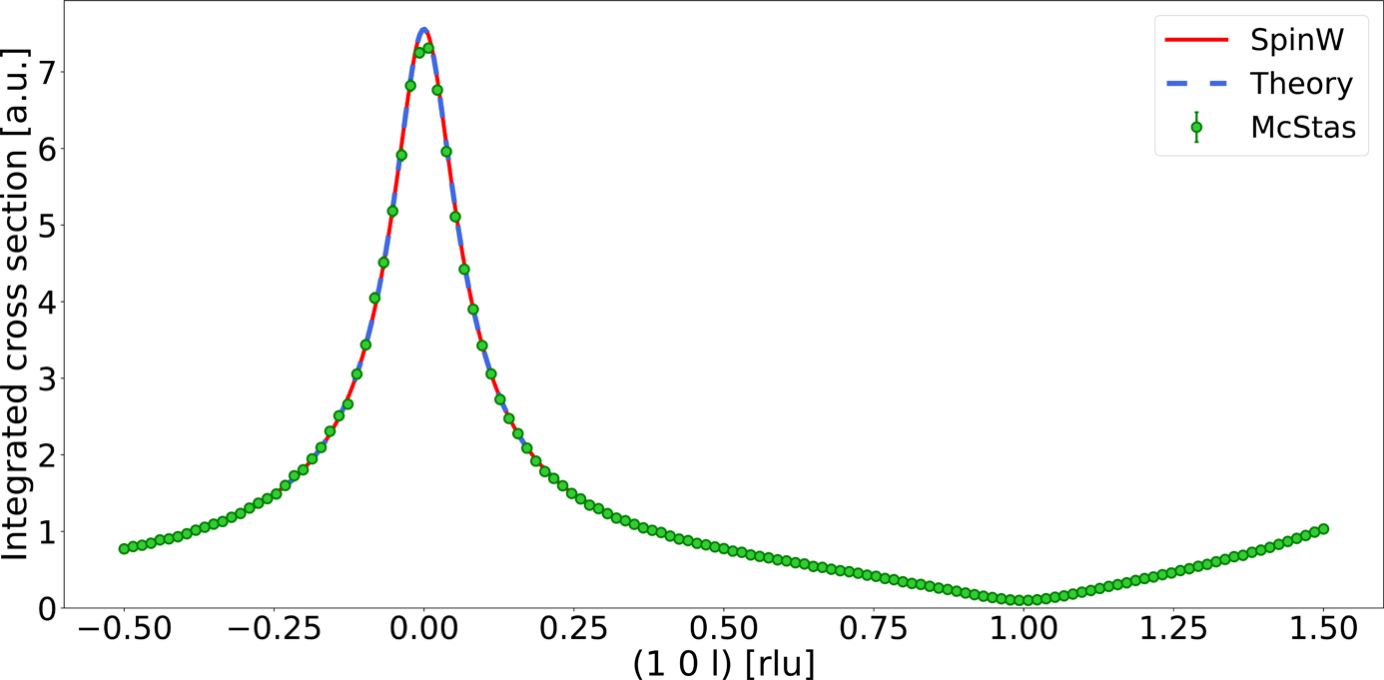
Comparison of integrated inelastic cross section between *McStas*, theory and *SpinW*. The theoretical model is shown to be compatible with the *SpinW* simulation. Data have been normalized to the 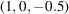 point.

The *SpinW* data were multiplied by the instrument-dependent factor 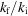, and this was then normalized to the 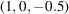 point of the *McStas* data for comparison. The two datasets can be seen in Fig. 6 ▸, where we also show that our model is compatible with the *SpinW* simulation. An excellent agreement is found, demonstrating that the SpinWave_BCO component is able, within a constant scaling factor, to accurately reproduce results obtained by *SpinW*.

The difference between the direct measurement of the differential cross section in Table 5 ▸ and the integrated cross section in Fig. 6 ▸ is a clear demonstration of the two distinctly different ways to integrate through reciprocal space: the differential cross section is an integration over 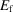 while keeping the scattering angle fixed, leading to the Jacobian, while Fig. 6 ▸ shows an integration over 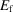, keeping 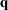 fixed, leading to data that are symmetric within a given Brillouin zone. As we demonstrate, these two equivalent methods both yield accurate results.

**Figure 7:**
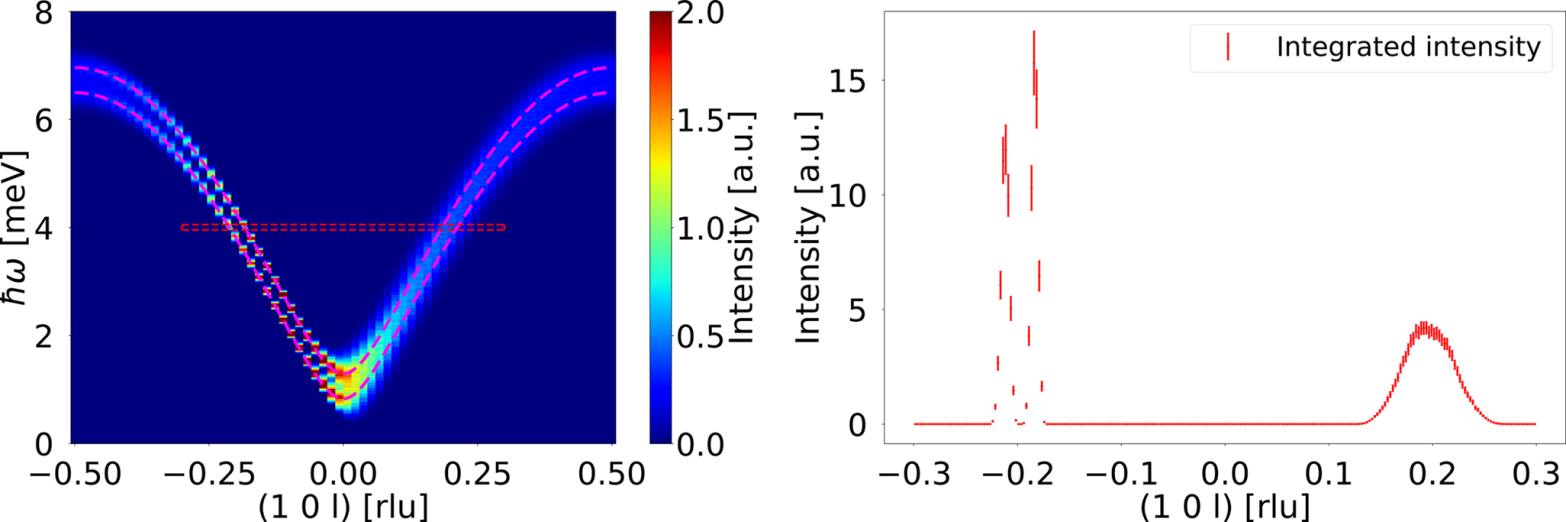
Result of simulations made with an applied magnetic field of 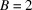
 T. (Left) The full 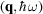 map showing all simulation data. (Right) A cut through these data at 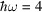
 meV and integrated along energy transfer with 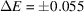
 meV (red dashed outline in the left panel).

To further demonstrate the capabilities of the component, a simulation was performed with an applied magnetic field of 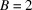
 T. The data are shown in Fig. 7 ▸. The two modes are split by the magnetic field, as expected. The magenta lines show the calculated spin wave mode dispersions, demonstrating that the simulated data again match well with theory. The area within the red outline was simulated using a **q** spacing six times smaller than the rest of the data. These data are shown integrated along the energy transfer on the right in Fig. 7 ▸. This clearly illustrates how the splitting is not always resolved. In this case this is due to the orientation of the resolution ellipsoid, leading to a focusing and a defocusing branch of the dispersion relation remeasurements simulated on the triple-axis spectrometer (Shirane *et al.*, 2002 ▸), which is also seen in Fig. 5 ▸. This effect is important to consider, even for our high-resolution TAS.

## 5. Altermagnetism in MnF_2_

As an additional functionality, we have implemented a simple altermagnetic dispersion. Altermagnetism in MnF_2_ was suggested by Šmejkal *et al.* (2022 ▸). While previous searches for the altermagnetic splitting of the magnon modes in MnF_2_ were unsuccessful (Morano *et al.*, 2025 ▸), a recent study revealed this splitting using polarized neutron scattering (Faure *et al.*, 2025 ▸). In the latter study, the spin-wave spectrum was modelled using both altermagnetic exchange couplings and long-ranged dipolar couplings. We will use a simpler model, only including the altermagnetic couplings, to show how the SpinWave_BCO component can be used for this purpose.

The theory of exchange-driven altermagnetic spin waves in MnF_2_ is described by McClarty *et al.* (2025 ▸). The lowest-order exchange interactions which lead to altermagnetic ordering are those between Mn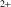 ions on the same sublattice at positions characterized by the vectors 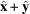 and 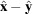, respectively. These coupling constants will be denoted 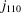 and 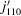, respectively. Including these in the Hamiltonian and performing the same diagonalization procedure as for the antiferromagnetic case leads to the altermagnetic dispersion relation. First, the Fourier transform of the coupling constants within each sublattice reads 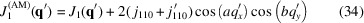
with 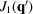 given by equation (12). Using the new 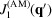, the spin wave dispersion becomes
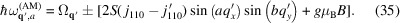
For 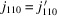, this reduces to the known antiferromagnetic result. For 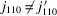 the two modes are split by the extra 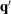-dependent term. This term is essentially added to the magnetic field, and just as the coherence factor is independent of the field, it is also independent of this term. Therefore, the intensity is essentially unchanged by the altermagnetic splitting. For this reason, we have implemented the altermagnetic splitting by simply modifying the dispersion relation according to equation (35). A simulation of the altermagnetic dispersion has been performed on the same TAS setup as used before.

**Figure 8:**
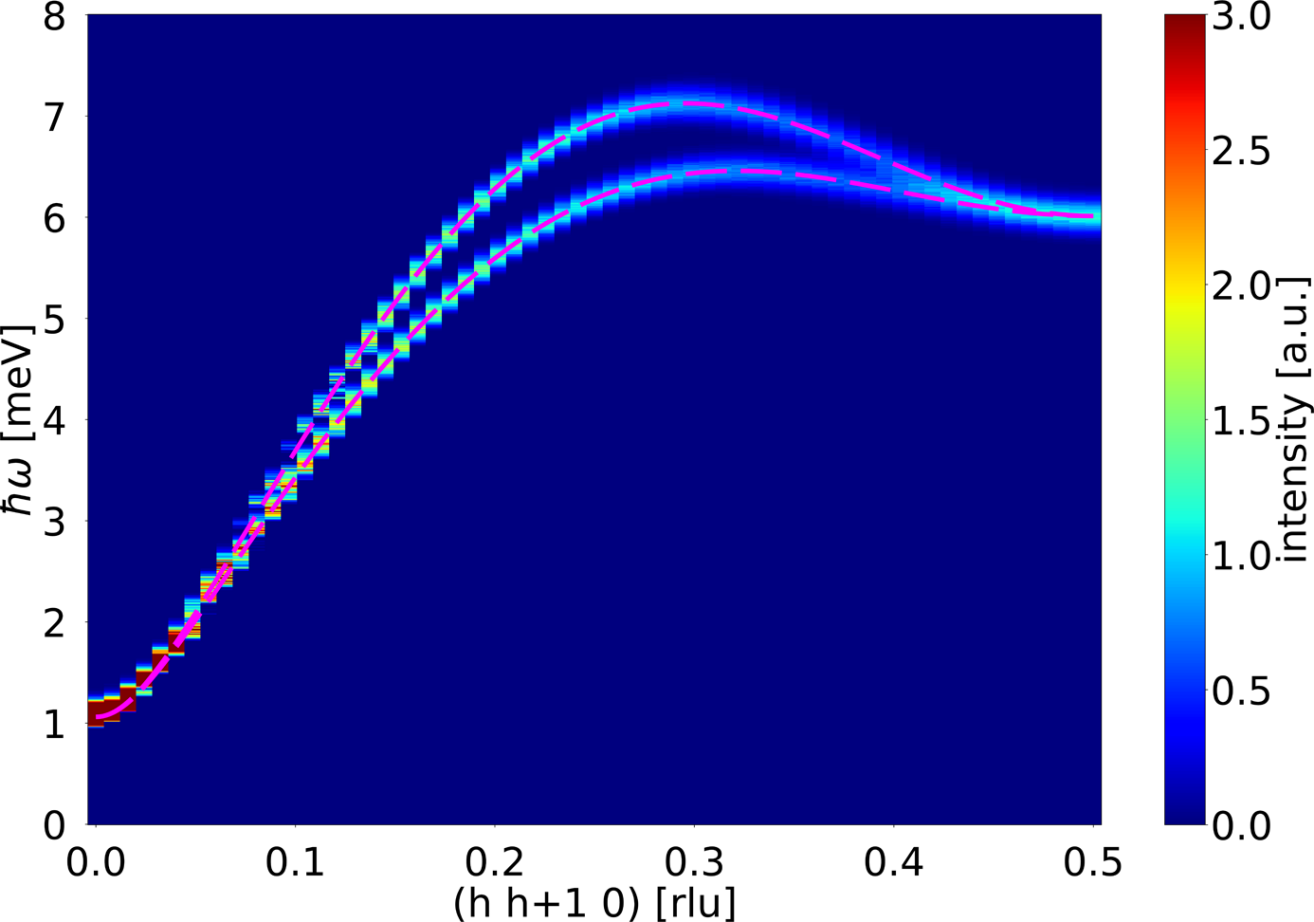
Simulated altermagnetic dispersion along 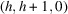. The dashed magenta lines show the theoretical dispersion relations for the split spin wave modes.

To show the splitting, the values of 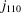 and 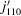 and the difference between them are made very large: 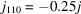 and 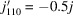. The simulated spectrum can be seen in Fig. 8 ▸, where the theoretical dispersion relation has been plotted for comparison. The altermagnetic splitting is clearly seen in the data.

## 6. Discussion and conclusion

We have demonstrated the implementation of accurate magnetic dynamics in *McStas* with a new sample component SpinWave_BCO, which simulates inelastic neutron scattering from spin waves in a ferromagnet or a two-sublattice antiferromagnet in a body-centred orthorhombic crystal structure. In addition, we have implemented and tested an altermagnetic splitting of the antiferromagnetic spin wave dispersion.

The simulated dispersion relation and differential cross section are found from linear spin wave theory. Simulated data have been compared directly with theory and with the program *SpinW* to verify the *McStas* simulations. This was exemplified by simulating the antiferromagnetic spin wave spectrum of MnF_2_. However, since the magnetic form factor is not taken into account, the simulated intensities are reliable only at small values of |**q**|.

We find that the simulated dispersions and absolute intensities agree extremely well with linear spin wave theory, both for magnon creation and for annihilation. This has been tested for many different values of |**q**|.

While our present work is an important step towards the description of magnetic dynamics in *McStas*, much development work lies ahead in this direction. The feature of greatest relevance will surely be to expand the description of lattice geometries and interactions to be able to model all crystal classes and also accommodate spin waves from *n*-sublattice systems and spiral order, in analogy with *SpinW* (Toth & Lake, 2015 ▸). To promote the calculation of realistic intensities, it could be of relevance to include a library of the magnetic structure factors, 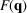, for the most common magnetic ions. It would also be relevant to evaluate other possible sampling methods, such as a pre-compiled grid of solutions to the kinematic constraint (26). A very ambitious, but also relevant, update would be to include a full description of neutron polarization, which is generally accommodated in *McStas* (Knudsen *et al.*, 2014 ▸) but the effect of which is not included in the SpinWave_BCO component.

## Figures and Tables

**Table 1:** Table of input parameters for the spin wave component SpinWave_BCO

Parameter name	Description
radius, yheight [m]	Radius and height of cylindrical sample.
sigma_inc, sigma_abs [barns]	Cross sections for incoherent scattering and absorption.
target_index	Relative index of component to focus at with next component being +1.
target_x, target_y, target_z [m]	Coordinates to focus at (alternative to target_index).
focus_xw, focus_yh [m]	Width and height of focusing.
focus_aw, focus_ah [°]	Direct specification of ΔΩ (alternative to focus_xw and focus_yh)
FM	Flag for type of order. Default is 0 (antiferromagnet), 1 selects ferromagnetic.
a, b, c [Å]	Lattice constants of orthorhombic lattice unit cell.
S	Spin of magnetic ions.
j [meV]	Magnetic interaction constant to the eight equivalent neighbours in the body-centred positions.
j_a, j_b, j_c [meV]	Magnetic interaction constants to the neighbours along the *a*, *b* and *c* axes, respectively.
j_110, j_110_prime [meV]	Magnetic interaction constants for altermagnetic splitting when FM = 0 (see Section 5). Both are 0 by default.
D [meV]	Uniaxial single-ion anisotropy constant.
B [T]	Magnetic field strength.
T [K]	Temperature.
mode_input	If FM = 0: Parameter to specify which mode is simulated. Value of 2 (default) simulates both modes. Value of 0 or 1 simulates only the corresponding mode.

**Table 2:** Input parameters to the spin wave component used in the validation process The antiferromagnetic parameters (*b*) match those modelled for the compound MnF_2_; adopted from Okazaki *et al.* (1964 ▸) and Nikotin *et al.* (1969 ▸).

(*a*)			(*b*)	
Ferromagnet			Antiferromagnet	
Parameter	Value		Parameter	Value
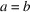	4.873 Å		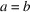	4.873 Å
*c*	3.130 Å		*c*	3.130 Å
*S*	2.5		*S*	2.5
*j*	−0.304 meV		*j*	0.304 meV
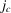	−0.056 meV		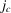	−0.056 meV
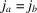	−0.008 meV		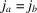	0.008 meV
*D*	−0.023 meV		*D*	−0.023 meV

**Table 3:** Ferromagnetic dispersion parameters obtained from a fit to constant-*q* scans, compared with the original values used in the simulation

Parameter	Value from fit [meV]	Input value to simulation [meV]
*j*	−0.303184 (8)	−0.304
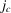	−0.05659 (3)	−0.056
*D*	−±0.0332 (1)	−0.023

**Table 4:** Antiferromagnetic dispersion parameters obtained from a fit to constant-*q* scans, compared with the original values used in the simulations

Parameter	Value from fit [meV]	Input value to simulation [meV]
*j*	0.30372 (1)	0.304
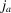	0.00800 (2)	0.008
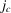	−0.05713 (2)	−0.056
*D*	−0.02359 (1)	−0.023

**Table 5:** Simulated (‘sim’) differential cross sections compared with results calculated from the analytic (‘ana’) expression at different points on the dispersion, defined by the point 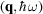 and index of the simulated mode Here Ψ_mon_ is the simulated flux at the monitor placed just before the sample, while *I*_det_ is the simulated intensity at the detector placed just before the analyser in the TAS setup.

*h*	*k*	*l*	ℏω [meV]	Mode	*B* [T]	Ψ_mon_ [cm^−2^ s^−1^]	*I*_det_ [s^−1^]	(dσ/dΩ)(sim) [cm^−2^]	(dσ/dΩ)(ana) [cm^−2^]
1	0	0	1.06	0	0	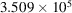	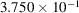	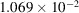	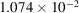
1	0	0	−1.06	0	0	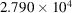	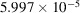	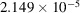	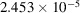
1	0	1	1.06	0	0	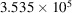	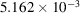	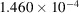	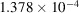
1	0	0.5	6.73	0	0	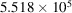	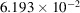	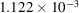	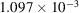
0.5	0	0.5	6.65	0	0	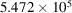	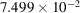	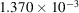	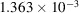
2	0	0.3	5.48	0	0	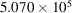	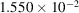	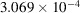	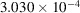
0.7	0	0.1	5.15	0	0	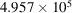	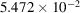	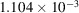	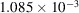
1	0	0	1.29	0	2	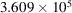	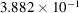	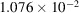	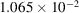
1	0	0	0.83	1	2	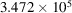	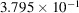	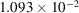	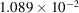

## Data Availability

All simulation files, simulated data and scripts for data analysis can be obtained from the authors upon reasonable request. The SpinWave_BCO component is included in the *McStas* component library, available from the homepage https://www.mcstas.org/.
